# Health Informatics: The Foundations of Public Health

**DOI:** 10.3390/healthcare11060798

**Published:** 2023-03-08

**Authors:** Tian-Shyug Lee, Chi-Jie Lu

**Affiliations:** 1 Graduate Institute of Business Administration, Fu Jen Catholic University, New Taipei City 242, Taiwan; 2 Artificial Intelligence Development Center, Fu Jen Catholic University, New Taipei City 242, Taiwan; 3 Department of Information Management, Fu Jen Catholic University, New Taipei City 242, Taiwan

## 1. Introduction

As technology continues to evolve, vast amounts of diverse digital data are becoming more easily generated and collected. In public health, various types of data can be generated. The vast volume and various types of data generated in public health, coupled with their characteristics such as value, variety, velocity, and veracity, present significant challenges in implementing appropriate techniques to deal with them [1,2]. To address these challenges, the field of health informatics has gained increasing popularity. Health informatics is a multidisciplinary field that focuses on designing, developing, and applying computational techniques to improve healthcare [2]. Given the wide variety of problems related to the use and handling of health data in public health, health informatics offers promising solutions through computational or machine learning techniques [3]. In this sense, health informatics can be considered the foundation of public health.

This Special Issue, entitled “Health Informatics: The Foundations of Public Health”, received 16 high-quality research articles. These articles explored the application of health informatics on public health-related problems.

## 2. The Organization of This Special Issue

In this Special Issue, the articles were sorted into three themes: Exploring Health Informatics with COVID-19 Data, Using Machine Learning for Public Health Insights, and the Application of Health Informatics in Public Health Management.

### 2.1. Exploring Health Informatics with COVID-19 Data

The COVID-19 pandemic, which began in 2019, has created a significant strain on healthcare systems globally. The pandemic has also generated extensive datasets related to COVID-19. The vast amount of COVID-19 data presents numerous health informatics research opportunities [4,5,6].

The rapid and contagious spread of COVID-19 has led to a surge in cases, causing a shortage of COVID-19 test kits in hospitals. To combat the spread of the virus and reduce the risk of infection, implementing an autonomous detection system is crucial. Therefore, in the study of Ayadi et al. [4], an automated system for COVID-19 detection and diagnosis was constructed. The COVID-19 detection function of their system was built with a neural network that concatenated AlexNet and Xception models. They named it COVID-AleXception, which was built to classify X-ray images for COVID-19 detection. COVID-AleXception achieved a promising performance with an accuracy of 98.68% and can help radiologists to diagnose COVID-19 faster.

Since the quality of the input dataset for constructing a model or performing an analysis will affect the overall reliability of the results, Binkheder et al. [5] discussed the data quality of COVID-19 patient records in their study. They evaluated the quality of the records and their readiness for secondary use. They demonstrated the existing shortcomings in documentation where a data quality assessment could be incorporated when using electronic health record data to ensure patient safety during documentation and to ensure data readiness for secondary use.

As governments and medical facilities manage public health during the COVID-19 pandemic, the number of confirmed cases plays an important role in policy and decision making. A dramatic increase in the number of COVID-19 cases without early information or notification may lead to a shortage of medical facilities and personnel, which may result in a disaster. Therefore, the study by Duangchaemkarn et al. [6] developed a seasonal autoregressive integrated moving average (SARIMA) model to predict the number of COVID-19 cases in Thailand. Their study presented the most accurate SARIMA model to forecast at least 28 days ahead of the current outbreak in Thailand, especially for the daily COVID-19 confirmed cases.

### 2.2. Using Machine Learning for Public Health Insights

The analysis of large amounts of data on a specific disease using health informatics techniques can provide valuable insights that can often benefit the community and support the development of guidelines and policies [7,8,9,10,11].

Analyzing important risk factors or trends related to a disease is often a common task in public health datasets. In the study by Jhou et al. [9], six well-known ML techniques, namely, random forest (RF), logistic regression (LGR), multivariate adaptive regression splines (MARS), extreme gradient boosting (XGBoost), gradient boosting with categorical features support (CatBoost), and a light gradient boosting machine (LightGBM), were used to construct an effective hybrid system for the evaluation of important risk factors in subjects with metabolic syndrome and stage 3 chronic kidney disease. Their results suggested that BUN (blood urea nitrogen), SBP (spontaneous bacterial peritonitis), right intraocular pressure (R-IOP), RBCs (red blood cells), and T-Cho/HDL-C (total cholesterol/high density lipoprotein cholesterol) were identified as important variables.

Tsai et al. [10] aimed to assess the effect of low-dose aspirin use in patients with predialysis advanced chronic kidney disease (CKD). In their study, a Cox regression model was used to estimate the hazard ratio and a machine learning method was used for feature selection to assess the importance of parameters in clinical outcomes. They found that aspirin use in patients with predialysis advanced CKD and anemia was associated with a higher risk of entering dialysis and death before entering dialysis during a 1.54-year follow-up. While nonusers and aspirin users did not significantly differ in renal effects, aspirin use was linked to a higher risk of bleeding, intracranial hemorrhage events, or ischemic stroke.

In the study by de Andres-Sanchez and Belzunegui-Eraso [11], complementary ordered logistic regression and fuzzy set qualitative comparative analysis were used to explore cannabis use among adolescents in Tarragona, Spain. The study found that being female, parental monitoring, and religiousness were significant inhibitors of cannabis consumption, while parental tolerance to substance use and having close peers that consume substances act as enablers.

### 2.3. Application of Health Informatics in Public Health Management

Management issues concerning individuals and organizations are integral to the research field of public health. By effectively utilizing health informatics techniques, valuable information can be extracted to provide deeper insights that support informed decision making [12,13,14,15,16,17,18,19].

Lin et al. [16] estimated the efficiency of 19 tertiary hospitals in Taiwan using data envelopment analysis and Tobit regression. They found that both the CCR (Charnes, Cooper, and Rhodes) and BCC (Banker, Charnes, and Cooper) models are suitable for evaluating hospital efficiency. This study also identified key factors that affect efficiency such as higher bed occupancy and fixed asset turnover rates, as well as areas for improvement in hospital performance such as surplus or deficit of appropriation, modified EBITDA (Earnings Before Interest, Taxes, Depreciation and Amortization), and self-pay income.

Pereira et al. [17] addressed the feasibility of updating and installing a hospital information system for the Brazilian Navy by using THOR 2 and PROMETHEE-SAPEVO-M1 methods to understand, structure, and clarify related variables. The study compared two methods (Commercial Software Purchase and Adoption of Free Software) for implementing a healthcare information system (HIS) and found that both were favorable for HIS feasibility. Additionally, the study emphasized the importance of discussing business perspective and changes in internal development in military healthcare management.

Lee et al. [18] conducted a study to examine the demographic characteristics and quality of life of patients with knee osteoarthritis and identified the demographic factors that affect their quality of life. They used a questionnaire survey and correlation analysis in their study. This study found a significant correlation between monthly disposable income and both physical and mental health components of quality of life (QOL) in patients with bilateral knee osteoarthritis, highlighting the crucial role of income as a factor affecting the QOL of these patients.

In the study by Chen et al. [19], the association between regular leisure time physical activity (LTPA) and self-reported body mass index and obesity risk among middle-aged and older adults in Taiwan was examined using multiple linear regression and logistic regression. They found that regular LTPA was associated with the male gender, normal weight, excellent or good self-reported health status, and a lower rate of being underweight compared to nonregular LTPA. Furthermore, regular LTPA was negatively associated with being underweight among middle-aged and elderly adults in Taiwan, but it had no significant relationship with BMI and obesity.
